# Fitting Assistive Technology for People with Hearing Loss: The Importance of Remote Microphone Systems′ Electroacoustic Verification

**DOI:** 10.3390/ijerph182413251

**Published:** 2021-12-16

**Authors:** Regina Tangerino de Souza Jacob, Elaine Cristina Moreto Paccola, Érika Cristina Bucuvic, Manoel Henrique Salgado

**Affiliations:** 1 Department of Speech-Language Pathology and Audiology, Bauru School of Dentistry, University of São Paulo, São Paulo 17012-901, Brazil; 2 Division of Hearing Health, Hospital for Rehabilitation of Craniofacial Anomalies, University of São Paulo, São Paulo 17012-901, Brazil; elainepaccola@usp.br (E.C.M.P.); erika.bucuvic@usp.br (É.C.B.); 3 Bauru Faculty of Engineering, Production Engineering Department, UNESP, São Paulo 17012-901, Brazil; henrique.salgado@unesp.br

**Keywords:** hearing assistive technology, remote microphone system, hearing loss

## Abstract

The remote microphone system (RMS) must be appropriately working when fitting it in a person with hearing loss. For this verification process, the concept of transparency is adopted. If it is not transparent, the hearing aid (HA) may not capture the user’s voice and his peers appropriately, or the RMS may not have the advantage in gain needed to emphasize the speaker’s voice. This study investigates the influence of the receiver’s gain setting on the transparency of different brands and models of RMS and HAs. It is a retrospective chart review with 277 RMS from three distinct brands (RMA, RMB, and RMC) and HAs. There was an association of the receiver’s gain setting with the variables: brand of the transmitter/receiver (*p* = 0.005), neck loop’s receiver vs. universal and dedicated receivers (*p* = 0.022), and between brands of HA and transmitter/receiver (*p* < 0.001). RMS transmitter (odds ratio [OR = 7.9]) and the type of receiver (neckloop [OR = 3.4]; universal [OR = 0.78]) presented a higher risk of not achieving transparency in default gain, confirming and extolling the need to include electroacoustic verification in the protocol of fitting, verification, and validation of RMS and HA.

## 1. Introduction

**Figure 1:**
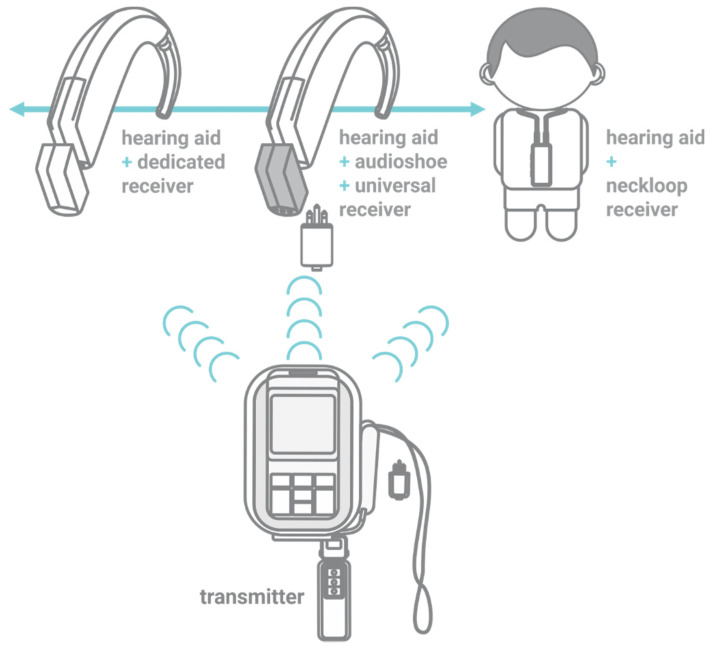
Remote microphone system: Transmitter and three types of receivers—dedicated, universal and neckloop.

The improvement in speech perception in noise for persons with hearing loss (HL) when using assistive technology, such as the remote microphone system (RMS), is well established [1]. The RMS consists of a transmitter that sends a signal to a receiver connected to a hearing aid (HA) (Figure 1). The audiologist should perform a combination of tests to fit it appropriately, including an electroacoustic evaluation and behavioral tests [2,3,4,5,6]. Even when the RMS and HAs are functioning correctly, the outcome may be compromised if the devices are not programmed to the user’s needs. The focus of this paper is not the behavioral tests, such as evaluation of speech perception in noise or questionnaires; we focus on RMS and HA’s electroacoustic evaluations.

Some exploratory studies on the characterization of the procedures used by audiologists to evaluate RMS found limited evidence on the use of electroacoustic measures [7], and several studies reinforce the importance of audiologists advocating the need for the use of these measures in the assessment of HA and RMS [8,9,10]. However, these studies were developed with restricted samples of HA and RMS, prioritizing the results of only one manufacturer [8,11]. Thus, the results were limited given the offers of different current models and technologies, such as the digital modulated (DM) system, which followed the predecessor frequency modulated (FM) system.

Initially, the researchers used the ANSI standards indicated for measuring HA to determine the electroacoustic characteristics of pure-tone signals of the FM system [12,13]. The results of these studies provided evidence that coupling an FM system to a HA, regardless of whether it occurred via an electromagnetic induction collar or direct audio input (DAI), would not guarantee that the electroacoustic characteristics of the HA determined by the ANSI standards were maintained [14].

The American Speech-Language-Hearing Association (ASHA) then published the first assessment guide specifically for FM systems [15,16], which was updated in 2002 [2]. Due to the technological advances applied to the RMS, other guidelines for best practices and standards were developed [4,5,6,17,18].

Currently, for the verification of the RMS’s electroacoustic characteristics, the concept of transparency is adopted. Nelson [19,20] and Platz [21] introduced the term when arguing that the audio signal’s transfer from the sound source into the FM system should ideally be fully transparent (i.e., the transfer should not change the frequency or amplitude characteristics of the captured signal).

The American Academy of Audiology defines transparency as the condition in which equal inputs to remote and local microphones generate equal outputs to the hearing device. The operational definition is that transparency is achieved when inputs of 65 dB SPL to the FM microphone produce an equal 65 dB SPL input to the HA microphone [4]. If the difference is >+2 dB, the RMS setting must be appropriately changed and re-evaluated to confirm transparency. For example, if the RMS average is 4 dB lower than the HA average, the RMS setting should be increased by 4 dB and the average differences recalculated.

The impact of transparency on RMS’s user performance is undisputed since, if it does not occur, the individual may not be able to monitor their own voice, listen to their peers properly, or benefit from the RMS advantage. However, there is a wide disparity between the time taken to adjust and evaluate HA compared to wireless technology, especially for the pediatric population. For example, a child may attend three or four initial consultations with an audiologist to fit, verify, and validate the amplification and, after that, the RMS may be fitted in a single consultation, without the proper time to fit it and verify the electroacoustic frequency response curve [9].

This paper aims to corroborate the impact of verifying the electroacoustic measurement on the RMS and HA’s fitting process and reinforce the importance of using standardized protocols in the rehabilitation process of individuals with HL.

The discussion about the effective use of the RMS can only be valid when all technical aspects of the operation are contemplated while fitting the device. Therefore, the hypothesis is that the electroacoustic measurement is necessary to evaluate the transparency and direct the need to adjust the RMS setting to achieve it, even in the face of technological advances applied to RMS and HA.

This research investigates whether the desired transparency in different brands and models of HA is achieved without adjustments in the RMS setting. Specifically, we aimed to answer two questions:

(1) Does the RMS setting require adjustment to achieve transparency?
(2) If the transparency is achieved without adjustments in the RMS setting, can it be associated with the degree of hearing loss, RMS’s and HA’s brand, or type of receiver?

## 2. Materials and Methods

### 2.1. Participants

This study is a retrospective chart review approved by the Institutional Review Board (CAAE 32128414.5.0000.5417; 32128414.5.3001.5441; 62585716.7.0000.5441 and 81825518.7.0000.5441).

The RMS database was composed of charts of 144 children and adolescents with mild to profound HL registered in a hearing service clinic. The analysis refers to the characteristics of the RMS and HA used by the patients; thus, the final sample is 277 RMSs from three different brands (RMA, RMB, and RMC) and HAs from seven different brands.

### 2.2. Procedure, Stimuli, and Equipment

To analyze the transparency data, we adopted the standard suggested by the Clinical Practice Guidelines: Remote Microphone Hearing Assistance Technologies for Children and Youth from Birth to 21 [4]. The transparency should not exceed the difference ≤±2 dB between the average output curves for frequencies 750, 1000, and 2000 Hz with 65 dB SPL digital speech input for the RMS and HA (local) microphones, except for the electromagnetic induction in neckloop receivers, in which the difference transparency should not exceed 5 dB [22].

**Figure 2:**
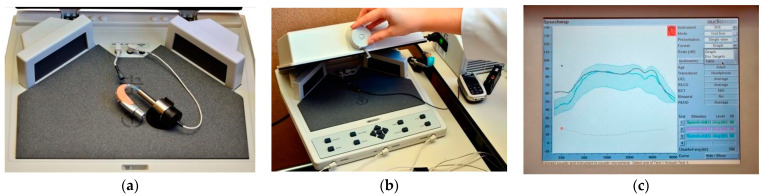
Set-up of electroacoustics measurement. (**a**) The HA plus RMS receiver inside the box; (**b**) the transmitter’s microphone inside the box; (**c**) frequency response curves for the HA and RMS.

Each procedure lasted about five minutes and consisted of two measurements with the 2cc coupler (one with the HA plus RMS receiver inside the box and another with the transmitter’s microphone inside the box, Figure 2). For neckloop receivers, the audiologist wore the neckloop and positioned the HA into the electromagnetic field outside the box, holding it in the same direction, next to the ear that the user wears it. The equipment generated information in multi-curve forms to evaluate how the HA was working in terms of amplification through frequencies. The audiologist wrote down the results in the patient’s chart. There was no reliability check since the data were collected during their routine appointment. The HAs were all tested with fresh batteries.

The electroacoustic measuring equipment with calibrated test box and 2cc coupler (models HA-2 and HA-1) were FP35 (Fonix) and Verifity (Audiscan) to FM systems. According to Auriemmo et al. [23], the transparency results from two equipment with different test boxes do not differ since they were calibrated and the process used similar speech stimuli.

Data analysis was performed using descriptive and inferential statistics. The variables are nominal and ordinal qualitative variables and were therefore described by relative frequency (percentage). Graphical representations were also presented according to these measures to illustrate results. For the association between two independent groups, the test was Chi-square(X²). A significance level of 5% (*p* = <0.05) was set for the inferential analyses. Odds ratio (OR) was calculated with a 95% confidence interval (95% CI) to analyze variables associated with the RMS default setting [24]. Excel (Microsoft, Redmond, Washington, DC, USA) and the software Minitab (Minitab, LLC, State College, PA, USA) were used.

## 3. Results

### 3.1. Does the RMS Setting Require Adjustment to Achieve Transparency?

The gain regulation necessary to make the device transparent was registered for each receiver model of the three brands of RM. The RMA had four receiver models (A1, A2, A3 and A4); the RMB had five receiver models (B1, B2, B3, B4 and B5), and the RMC had two models (C1 and C2).

**Figure 3:**
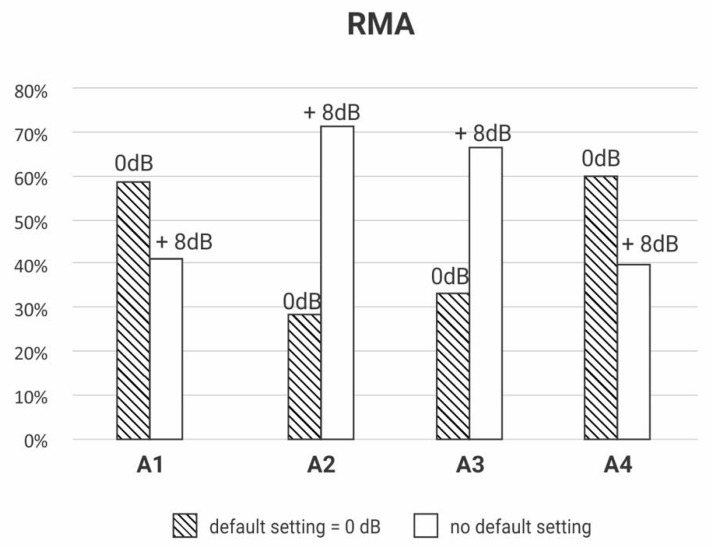
Descriptive analysis of RMA’s receivers’ (A1, A2, A3, A4) gain setting to achieve transparency (*n* = 80).

**Figure 4:**
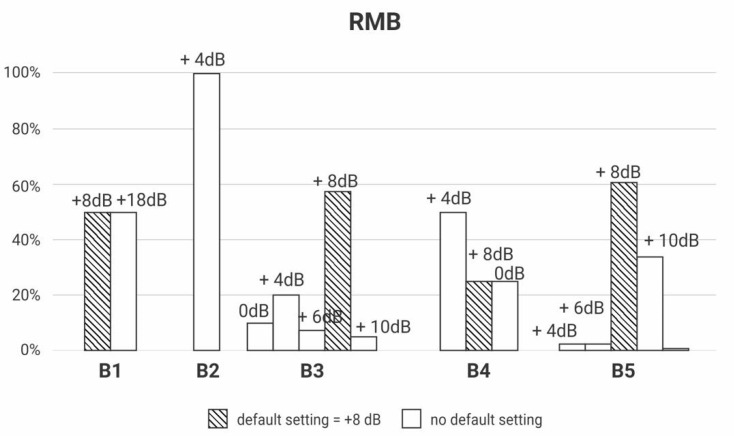
Descriptive analysis of RMB’s receivers’ (B1, B2, B3, B4, B5) gain setting to achieve transparency (*n* = 183).

**Figure 5:**
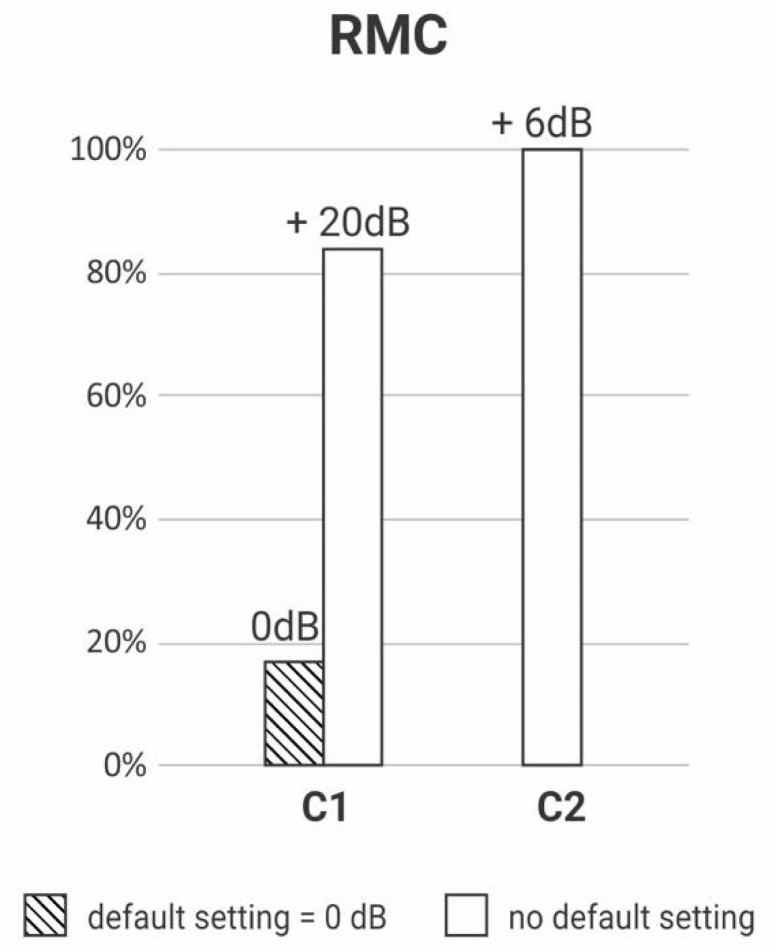
Descriptive analysis of RMC’s receivers’ (C1, C2) gain setting to achieve transparency (*n* = 14).

Figure 3, Figure 4 and Figure 5 depict the percentage of gain settings for each receiver model of one of the brands (RMA, RMB, RMC) to achieve transparency. All the RMS were transparent, but the gain setting default mode was not always enough to achieve it; some receivers needed different gain settings adjustments.

The Y-axis of Figure 3, Figure 4 and Figure 5 show the percentage of default gains and different gain adjustments. For RMA and RMC, the default gain is 0 dB; for RMB, the default gain is 8 dB; the default gains are streaked on Figure 3, Figure 4 and Figure 5. Any adjustment value different from the default gain was considered “no default setting”.

### 3.2. If the Transparency Is Achieved without Adjustments in the RMS Setting, Can It Be Associated with the Degree of Hearing Loss, RMS’s and HA’s Brand, or Type of Receiver?

Results in Table 1 suggest an association of the default setting with the transmitter/receiver brand (*p* = 0.005) and with a receiver (when comparing universal, design-dedicated and induction neckloop) (*p* = 0.022).

There was a difference in the odds ratio of the default setting between RMB and RMC transmitters/receivers (*p* = 0.002); between design-dedicated and universal receivers (*p* = 0.030); and induction neckloop and universal/design-dedicated receivers (*p* = 0.020). In addition, there was a higher probability of the default setting in RMB transmitters (between brands, but without difference between RMA and RMB), design-dedicated receiver and less likely in induction neck loop receiver.

## 4. Discussion

The main contribution of this study is confirming the need to make adjustments to the RMS setting in different models and brands to achieve transparency (Figure 2, Figure 3 and Figure 4).

There are previous studies that research RMS’s use. These studies examined the teachers’ perspective and the family’s involvement with the consistency of RMS’s use by school children. They usually describe the analysis of charts, fit RMSs and handle initial guidelines; however, they do not inform the verification protocol used [25,26].

In addition, wireless technology coupling to the HA may result in some undesirable gain/output changes depending on the neckloop arrangements and the DAI. Equally problematic is the variability in acoustic output that can occur between wireless technology configurations. These findings concluded that HA technology requires more than a cursory check; consequently, there have been efforts to develop protocols that practitioners can use to evaluate and verify wireless technology for individuals with HL [4,9,10].

The present study adopted the American Academy of Audiology guide [4] to calculate transparency. Mulla [11] compared this protocol with those proposed by UKCFMWG (2008) [6] and iPOP (2009) [17] by evaluating 14 Phonak design-dedicated receivers (ML10, 11 and 12i) with the Inspiro transmitter and three Phonak HA models (Naída UP, Naída SP and Nios). The author observed that, according to the AAA and iPOP criteria, the gain of two devices needed to be adjusted to achieve transparency with the Nios model and two devices of this model did not. However, the two devices that needed adjustments would be transparent by the UKCFMWG guide, which recommends a higher number of frequencies for calculating transparency.

The value considered as the limit for correcting the response curve overlap is also not a consensus in the literature, ranging between ±/≤2 dB (AAA, 2011; UKCFMWG, 2008; iPOP 2009; PHONAK, 2016) [4,6,17,18], 3 dB [27] to 5 dB [22].

In another study [28], when evaluating the Inspiro transmitter with the Mlxi receiver (universal) and Phonak’s Nathos HA (corresponding to Naída), transparency was achieved using the default settings. However, in other studies [8,11], the HA Nios (Phonak) also required a change in the gain of the Mlxi (universal) receiver by –6 dB to achieve transparency. Therefore, we emphasize that clinicians must record the RMS gain data in the patient’s chart, even if the value can be accessed directly on the device during the follow-up consultation.

In this study, besides observing the adjustments of the frequency response curves of HAs and altering the RMS’s gain setting to achieve transparency, we showed the difference of occurrence distribution between them and the influence of the brand and model of RMS and type of receiver (Table 1). Furthermore, the above studies cited evaluated HA and FM System of only one manufacturer, while we evaluated three different brands and neckloop induction receivers.

In clinical practice, it is usually unnecessary to set the gain to achieve transparency when the whole set is of the same brand, and the receiver is design-dedicated. Two studies corroborate the results shown in Table 1: the one [11] with Phonak’s HA and FM in this condition and another one [10] in which the FM gain was set to +12 dB to achieve transparency with the T20 Transmitter and Vigo Pro C with the Amigo R12 receiver (design-dedicated). Both studies showed a higher chance of occurrence in the design-dedicated type, but not all receivers achieved transparency with the default gain.

However, a portion of those RMS in the default setting is expected to be transparent, as indicated in Figure 2, Figure 3 and Figure 4 [8,29].

In Table 1, with the transmitter/receiver analysis, we observe that, on the one hand, the frequency of need for gain setting and use of default setting was similar between the RMB and RMA brands; on the other hand, RMC had a higher frequency of need for gain setting. In studies involving the verification of electroacoustic measurements, the authors cite that when transparency values are not achieved with the default setting, they should be adjusted using the receiver gain setting controls that can be activated by the transmitter or the company’s software [3,9,11,14,23,30].

The possibility of controlling the programming of the direct audio input (DAI yes or no) by the HA company software also interferes in the relative impedance of the RMS signal resulting from each combination of these definitions, which the audiologist should analyze for each model of HA and RMS receiver. This characteristic probably influenced the association between the HA brand variable and the transmitter and receiver brand variables (Table 1) [31].

Frequency response curve analysis is required to confirm that the entire system (HA and RMS) is configured and working correctly. That ensures the user receives a clear signal from the speaker using the RMS microphone while still listening to classmates and oneself with the local microphone, the prevailing situation in the current educational environment [3,21,32,33]. The studies cited above also emphasize the importance of RMS electroacoustic verification in determining the necessary modifications and thus providing a better fit for the patient. When adequately verified, the user’s audibility and speech recognition in noise with RMS should be as good as under ideal listening conditions.

It is noteworthy that, in the cases of younger children whose language is still developing and who cannot objectively report the device’s benefit, the electroacoustic data may be the only indication of the effective functioning of the FM system [11]. Nevertheless, the benefit of RMS in early childhood for language acquisition and development is already strongly evidenced by the literature [11,34,35,36,37,38].

In this study, we found great variability between brands and models regarding the need to set the gain of these devices, confirming the importance of measuring the transparency in the RMS selection routine (Figure 2, Figure 3 and Figure 4). This standardization of clinical conduct becomes relevant mainly in hearing services provided by the Brazilian Public Health System, where devices are usually purchased by bidding, generating a great rotation of brands and models of HA and RMS for concession.

For either public or private clinics, the pursuit of certification of service quality for RMS fitting is necessary to improve the quality of the service provided, aiming for effective use of the device in daily activities and avoiding the waste of public funds.

If transparency does not occur, the HA’s microphone may not adequately capture the student’s and his colleagues’ voices, impairing their acoustic-articulatory feedback, interaction, and inclusion in the classroom. Another possible consequence is when the RMS provides a superior amplification of the teacher’s voice compared to the HA’s amplification of coexisting sounds.

The technology employed in RMS development is advancing rapidly, and new products and features continue to be introduced to the market. Therefore, audiologists need to advocate for electroacoustic evaluation of all devices independently and consistently to verify and evaluate the reliability of these advanced features [9]. The need for manufacturers to adopt a common standard for RMS design and technology is supported by the ANSI/ASA S3.47 (2014) standard [39]. It addresses specific measures that analyze electroacoustic characteristics, making it easier to compare between makes and models of devices and determine if they meet the specifications described. We contacted sales representatives to discover some of the specifications of the devices that were evaluated in this study, as they were not found in the available datasheets.

The purpose of this research was to reinforce the need for electroacoustic evaluation of the RMS. However, the clinician cannot expect this setting to always result in improved performance for the patient. It is the initial stage of fitting RMS; however, it does not exclude the need to assess speech perception in noise, evaluate the benefit and satisfaction, and counsel and train users, caregivers, and teachers on the wireless technology.

## 5. Conclusions

The results obtained in this study led to the conclusion that the default RMS setting gain was not sufficient to guarantee transparency in all brands and models of RMS and HA evaluated. Furthermore, the type of receiver and the model and brand influenced the adjustment of the RMS gain setting, confirming and emphasizing the need to include electroacoustic verification in the protocol of fitting, verification, and validation of RMS and HA.

## Figures and Tables

**Table 1:** Analysis of the association between the default setting variable and the degree of hearing loss (HL), transmitter/receiver brand, type of receiver, and hearing aid (HA).

Variable	Category		Default Setting		*p*-Value	OR	CI _95%_	*p*-Value
			Yes	No				
Degree of hearing loss	Mild	*n*	6	10	0.264			
		%	4.1	7.6				
	Moderate	*n*	59	47		0.48	0.16–1.41	0.180
		%	40.4	35.8				
	Severe	*n*	52	39		0.45	0.15–1.34	0.180
		%	35.6	29.7				
	Profound	*n*	29	35		0.72	0.23–2.23	0.570
		%	19.8	26.7				
Transmitter/receiver brand	RMB	*n*	104	79	0.005 *			
		%	71.2	60.3				
	RMA	*n*	40	40		1.32	0.78–2.23	0.310
		%	27.4	30.5				
	RMC	*n*	2	12		7.90	1.72–36.32	0.002 *
		%	1.3	9.1				
Receiver	Design-dedicated	*n*	110	97	0.053			
		%	75.3	74.0				
	Universal	*n*	32	22		0.78	0.42–1.43	0.030 *
		%	21.9	16.7				
	Neckloop	*n*	4	12		3.40	1.06 to 10.90	0.420
		%	2.7	9.1				
Receiver	Design-dedicated	*n*	110	97	0.421			
		%	77.4	81.5				
	Universal	*n*	32	22		0.78	0.24–2.50	0.420
		%	21.9	16.7				
Receiver	Neckloop	*n*	4	12	0.022 *			
		%	2.7	9.1				
	Universal and design-dedicated	*n*	142	119		0.28	0.09–0.89	0.020 *
		%	97.2	90.8				
HA	RMB	*n*	79	65	0.241			
		%	54.1	49.6				
	RMA	*n*	47	38		0.98	0.57–1.68	0.940
		%	32.1	29				
	Other	*n*	20	28		1.70	0.88–3.30	0.110
		%	13.7	21.3				

*: Statistical significance
